# Effect of calcitonin gene-related peptide (-receptor) antibodies in chronic cluster headache: Results from a retrospective case series support individual treatment attempts

**DOI:** 10.1177/0333102420949866

**Published:** 2020-08-17

**Authors:** Ruth Ruscheweyh, Gregor Broessner, Gudrun Goßrau, Katja Heinze-Kuhn, Tim P Jürgens, Katharina Kaltseis, Katharina Kamm, Andreas Peikert, Bianca Raffaelli, Florian Rimmele, Stefan Evers

**Affiliations:** 1 Ludwig Maximilians University Munich, Department of Neurology, Munich, Germany; 2 Headache Outpatient Clinic, Department of Neurology, Medical University of Innsbruck, Innsbruck, Austria; 3 Headache Outpatient Clinic, Interdisciplinary Pain Center, University Hospital and Faculty of Medicine Carl Gustav Carus, TU Dresden, Dresden, Germany; 4 Migraine and Headache Centre, Pain Clinic Kiel, Kiel, Germany; 5 Headache Center North-East, Department of Neurology, University Medical Center Rostock, Rostock, Germany; 6 Neurologicum Bremen Outpatient Center for Neurology and Psychiatry, Bremen, Germany; 7 Charité Universitätsmedizin Berlin, Department of Neurology, Berlin, Germany; 8 Department of Neurology, Krankenhaus Lindenbrunn, Coppenbrügge, Germany; 9 Faculty of Medicine, University of Münster, Münster, Germany

**Keywords:** Chronic cluster headache, preventive treatment, headache diary, galcanezumab, erenumab, CGRP

## Abstract

**Objective:**

To assess the efficacy of monoclonal antibodies targeting calcitonin gene-related peptide (CGRP) or its receptor in chronic cluster headache (CCH) treatment under real world conditions.

**Background:**

Calcitonin gene-related peptide has an important pathophysiological role in cluster headache. Although the randomised controlled trial with the calcitonin gene-related peptide antibody galcanezumab was negative, chronic cluster headache patients with insufficient response to other preventive treatments have been receiving individual off-label treatment attempts with calcitonin gene-related peptide-(receptor) antibodies.

**Methods:**

Data from 22 chronic cluster headache patients who received at least one dose of a calcitonin gene-related peptide(-receptor) antibody and recorded attack frequency in a headache diary were retrospectively collected at eight headache centres.

**Results:**

The number of previous preventive therapies was 6.5 ± 2.4 (mean ± standard deviation, range: 2–11). The average number of attacks per week was 23.3 ± 16.4 at baseline and significantly decreased by −9.2 ± 9.7 in the first month of treatment with a calcitonin gene-related peptide(-receptor) antibody (*p* < 0.001). Fifty-five percent of the patients were 50% responders and 36% were 75% responders with respect to attack frequency. Significant reduction of attack frequency started at week 1 (−6.8 ± 2.8 attacks, *p* < 0.01). Results were corroborated by significant decreases in weekly uses of acute headache medication (−9.8 ± 7.6, *p* < 0.001) and pain intensity during attacks (−1.2 ± 2.0, numerical rating scale (NRS) [0–10], *p* < 0.01) in the first month. In months 2 (n = 14) and 3 (n = 10), reduction of attack frequency from baseline was −8.0 ± 8.4 (*p* = 0.004) and −9.1 ± 10.0 (*p* = 0.024), respectively.

**Conclusion:**

Under real-world conditions, individual treatment with calcitonin gene-related peptide(-receptor) antibodies was effective in 55% of our chronic cluster headache patients. This finding supports individual off-label treatment attempts with calcitonin gene-related peptide-(receptor) antibodies in chronic cluster headache patients insufficiently responding to other therapies.

## Introduction

Cluster headache is characterised by excruciatingly painful side-locked headache attacks with ipsilateral cranial autonomic symptoms and restlessness, often recurring several times a day. It has a prevalence of ∼0.1%, a male preponderance, and is one of the primary headache disorders associated with the highest disability, especially in its chronic form (1–3). Chronic cluster headache (CCH) affects 10–15% of cluster headache patients and is defined by attacks ongoing for ≥1 year, with attack-free periods lasting <3 months (4,5). Mainstays of long-term preventive treatment in Europe are verapamil, lithium and topiramate, complemented by neuromodulatory approaches and other drugs with less evidence (6,7). While these treatments work well for many patients, there is a relevant proportion that does not respond sufficiently, or does not tolerate treatment (8). There clearly is an unmet need for new therapies in CCH.

Similar to migraine, calcitonin gene-related peptide (CGRP) plays an important role in cluster headache pathophysiology (9). CGRP levels in jugular blood are elevated during active episodes between attacks, further elevated during attacks and reduced after successful acute treatment with oxygen or triptans, which act in part by inhibiting CGRP release from trigeminal nerve fibers (10,11). In addition, CGRP infusion induces attacks in active cluster headache patients (12). Antibodies inhibiting the activity of CGRP or its receptor (CGRP(R) antibodies) are effective in migraine and exhibit a favourable safety profile (13). The CGRP antibody galcanezumab has been tested in cluster headache in two randomised, placebo-controlled clinical studies. In episodic cluster headache, a significant reduction of attack frequency was found, leading to approval by the US Federal Drug Administration (FDA) in June 2019 (14). The effect was not significant in CCH, potentially due to a large placebo or regression to the mean response (15). The European Medicines Agency (EMA) declined approval of galcanezumab for treatment of cluster headache in February 2020.

Nonetheless, for CCH patients refractory to other preventive therapies and severely affected by high frequencies of attacks going on for months, an individual treatment attempt with a CGRP(R) antibody appears to be a therapeutic option with a convincing pathophysiological rationale. With the availability of the CGRP(R) antibodies on the European market in 2018 and 2019, headache centres have started to provide individual off-label treatment attempts with CGRP(R) antibodies to selected CCH patients. Off-label treatment is usual practice in CCH treatment, as controlled trials in this patient group are scarce.

While an open case series cannot provide information about the difference from placebo, it can help in estimating whether CCH patients can benefit from CGRP(R)-antibody treatment under real-world conditions. Therefore, we report on 22 cases of CCH treated with a CGRP(R)-antibody for at least 1 month and compared their weekly attack frequencies before and after treatment based on headache diary data. Our primary endpoint was the reduction of number of attacks in weeks 1–4 after CGRP(R) application with respect to the 4-week baseline. Where available, pain intensity and use of abortive treatment were also analysed.

## Methods

### Patients

This is a retrospective case series based on headache diary data of CCH patients, conceived during a meeting on the role of CGRP in headache organised by the German Migraine and Headache Society (DMKG) in February 2020. It includes adult (≥18 years old) patients diagnosed with chronic cluster headache (CCH) according to the International Classification of Headache Disorders, 3rd edition (ICHD-3) criteria (4), who received at least one treatment with a CGRP(R) antibody between December 2018 and March 2020, who did not change their concomitant cluster headache preventive therapy during the observation period (4-week baseline and months 1–3 as applicable), with one exception (discussed below), and who documented the frequency of their cluster headache attacks in a headache diary as part of their standard care. The decision to treat a patient with a CGRP(R) antibody was up to the clinical judgement of the treating physician, there were no standardised criteria. Patients who had received a CGRP(R) antibody within a clinical study were not eligible. We explicitly asked all participating centres to report all their cases fulfilling these inclusion criteria, irrespective of their response to the CGRP(R) antibody, to minimise selection bias. Seven German and one Austrian headache centre contributed data as follows: Berlin one patient, Bremen one, Coppenbrügge four, Dresden three, Innsbruck three, Kiel two, Munich four, Rostock four; total: 22 patients. There were an additional four patients, all male (one from Kiel, two from Innsbruck, one from Munich) who were treated with a CGRP(R) antibody within the recruitment period, but who did not document the frequency of their headache attacks or did not provide the documentation despite repeated requests.

Research was conducted according to the declaration of Helsinki. As this was a purely retrospective, fully anonymised analysis of data obtained by chart review after standard clinical care, approval from the ethics committee was not needed according to German and Austrian regulations.

A 4-week baseline and 1–3 months under continued treatment with the CGRP(R)-antibody were analysed, as available. All centres used very simple paper-and pencil headache diaries, collecting information on daily number of attacks, and some additionally on pain intensity during the attacks and on the use of cluster-headache specific acute headache medication (triptans or oxygen). Demographic data, and headache and treatment characteristics, were extracted from the patients’ charts (Table 1 and 2). Refractory CCH was defined by the criteria of the European Headache Federation (8).

**Table 1.** Characteristics of the study population (n = 22).

Age (years)	46.6 ± 12.3
Gender	15 female (68%)
Duration of cluster headache (years)	12.4 ± 7.3
Duration of chronic cluster headache (years)	6.6 ± 6.0
Primary chronic cluster headache^$^	9 (41%)
Affected side	7 right, 12 left, 3 alternating^$$^
Comorbid migraine	6 (27%): 3 CM, 3 EM
Current acute treatment	
Oxygen	15 (68%)
Sumatriptan 6 mg s.c.	13 (59%)
Sumatriptan 3 mg s.c.	4 (18%)
Zolmitriptan 5 mg i.n.	11 (50%)
Other^§^	3 (14%)
Current preventive treatment	
Verapamil	17 (77%), dose: 455 ± 263 mg
Lithium	2 (9%), dose: 563 ± 159 mg
Topiramate	6 (27%), dose: 133 ± 61 mg
Other^§§^	9 (41%)
Number of current preventive treatments	1.6 ± 0.9 (range: 0–4)
Previous preventive treatment	
Verapamil	21 (95%); IE 21, IT 10, CI 1 max. dose: 710 ± 232 mg
Lithium	16 (73%); IE 15, IT 11, CI 1 max. dose: 840 ± 365 mg
Topiramate	19 (86%); IE 16, IT 13, CI 0 max. dose: 144 ± 74 mg
Total number of previous preventive treatments^§§§^	6.5 ± 2.4 (range: 2–11)

Mean ± SD or numbers of patients and percentages are given.

IE: (number of patients with) insufficient efficacy of the drug; IT: (number of patients with) insufficient tolerability of the drug; CI: (number of patients with) contraindications for the drug; CM: chronic migraine; EM: episodic migraine.

Note: Ethnicity was white (Caucasian) for all patients.

^$^Primary chronic cluster headache means chronic cluster headache that did not evolve from episodic cluster headache.

^$$^Sides were alternating every few weeks to months, not from attack to attack.

^§^Other acute treatments were: Stimulation of the sphenopalatine ganglion, oral sumatriptan, opioids (injected/oral), diazepam.

^§§^Other current preventive treatments were: Candesartan (2), prednisolone (2), carbamazepine (2), deep brain stimulator (1), amitriptyline (1), naratriptan bid (1).

^§§§^ other previous preventive treatments were: Corticoids (18), onabotulinumtoxinA (13), oral or nasal triptans bid (9), greater occipital nerve block (9), non-invasive cervical vagus nerve stimulation (9), valproic acid (4), tricyclic antidepressants (4), pregabaline/gabapentine (4), stimulation of the sphenopalatine ganglion (3), indomethacin (3), melatonin (2), candesartan (2), occipital nerve stimulation (1), ergotamine (1), caffeine (2), levetiracetam (1), pizotifen (2), gamma-knife surgery (1).

**Table 2.** Description of CGRP(R) antibody treatment (n = 22).

Treatment started with	Galcanezumab 240 mg^$^	16 (73%)
	Erenumab 70 mg^$$^	3 (14%)
	Erenumab 140 mg	3 (14%)
Months under treatment until now		4.6 ± 4.3 (range: 1–16)
Observation period under treatment within present study		Month 1: 22 patients
		Month 2: 14 patients
		Month 3: 10 patients
Days between first and second treatment		31.0 ± 4.3
Days between second and third treatment		30.9 ± 2.8

^$^Was reduced to 120 mg in subsequent months in two patients.

^$$^Was increased to 140 mg in subsequent months in all patients and changed to galcanezumab 240 mg in the third month in one patient.

One of the patients taking prednisolone as a preventive treatment throughout the observation period adapted the daily prednisolone dose between 10 and 75 mg according to attack severity and frequency. Unfortunately, the patient did not record the prednisolone dose on a daily basis. However, this had been his practice for more than 6 months before starting the CGRP-antibody treatment, and it had been insufficient to control his attacks. In addition, he had been able to reduce the prednisolone dose from 40–75 mg daily before the start of the CGRP(R) antibody treatment to 10 mg daily after the second administration of CGRP(R) antibody (3 months were recorded for this patient).

### Endpoints, data extraction and missing data

For the purpose of the present analysis, month 1 was defined as weeks 1–4 after the first treatment with a CGRP(R) antibody, month 2 as weeks 5–8 and month 3 as weeks 9–12. Baseline refers to the 4 weeks preceding the first CGRP(R) antibody treatment.

Our primary endpoint was the reduction of number of attacks in month 1 with respect to baseline. Secondary endpoints were reduction of number of uses of acute medication and pain intensity during attacks in month 1 compared to baseline.

The number of cluster headache attacks per week, and, if available, the number of acute medication uses per week and the average pain intensity during attacks per week were extracted from the headache diaries. Pain intensity was assessed on a numerical rating scale (NRS) from 0 (no pain) to 10 (strongest pain imaginable).

We included only patients with a complete headache diary covering at least 4 weeks after the first CGRP(R) antibody administration, ensuring we had complete data from all 22 patients for the primary endpoint analysis. For months 2 and 3, data on attack frequency were available for 14 and 10 patients, respectively. This was due to several causes, including lack of continued use of a headache diary after the first month (four patients), discontinuation of treatment either because of lack of effect (one patient after first month, three patients after second month) or due to declined or delayed coverage of treatment costs by the patient’s health insurance (three patients after the first month, one patient after the second month). In addition, not all patients recorded the number of uses of acute headache medication and pain intensity. Numbers of patients available for each analysis are included in Table 3.

### Statistics

Statistical analysis was performed with the Statistical Package for Social Sciences version 25 for Windows (IBM, Armonk, NY, USA). *p* < 0.05 was considered significant (two-sided).

The following outcome parameters were analysed: Number of attacks per week, number of acute medication uses per week, average pain intensity during attacks per week.

For analysis of differences in number of attacks per week between baseline and month 1 (primary outcome), Wilcoxon’s test was used. The same procedure was used to compare the number of uses of acute medication and pain intensity during attacks between baseline and month 1 (secondary outcomes).

For analysis of outcome parameters on a weekly basis over the first 4 weeks, one-way repeated measures analysis of variance (ANOVA) was used with time as factor (baseline, week 1, week 2, week 3, week 4). Wilcoxon tests were used as *post-hoc* tests to compare baseline to each week, followed by Bonferroni-Holm correction for four comparisons.

A responder was defined as a patient having an average reduction of weekly attack frequency in month 1 of ≥50% with respect to baseline.

For analysis of the association of response with selected factors (age, gender, duration of cluster headache in years, total number of previous preventive treatments, and number of attacks per week at baseline), Spearman’s correlation or Mann-Whitney U tests were used as appropriate, followed by Bonferroni-Holm correction for five comparisons. For analysis of differences between baseline and months 2 and 3, pairwise Wilcoxon tests were used, followed by Bonferroni-Holm correction for each outcome parameter (three comparisons). Cohen’s d was used as a measure of effect size.

## Results

A total of 22 CCH patients fulfilled the inclusion criteria. Patients’ characteristics are listed in Table 1 and CGRP(R) antibody treatment characteristics are summarised in Table 2. Seventeen patients were treated off-label. In five patients, treatment was initiated because of comorbid migraine. Patients had received 6.5 ± 2.4 (range: 2–11) previous prophylactic treatments. The criteria for refractory CCH as defined in (8) were fulfilled by 19 patients.

**Table 3.** Effect of treatment with a CGRP(R) antibody in chronic cluster headache.

	Baseline	Treatment (change from baseline)		
		Month 1	Month 2	Month 3
Number of attacks per week	23.3 ± 16.4 (22)	**−9.2 ± 9.7 (22)** **d_z_ = 0.95** **Z = −3.3, *p* < 0.001**	**−8.0 ± 8.4 (14)** **d_z_ = 0.95** **Z = −2.9, *p* = 0.004**	**−9.1 ± 10.0 (10)** **d_z_ = 0.91** **Z = −2.3, *p* = 0.024**
Number of acute medication uses per week	16.2 ± 9.9 (19)	**−9.8 ± 7.6 (19)** **d_z_ = 1.30** **Z = −3.7, *p* < 0.001**	**−7.9 ± 7.5 (13)** **d_z_ = 1.06** **Z = −3.2, *p* = 0.001**	**−9.2 ± 8.0 (10)** **d_z_ = 1.15** **Z = −2.7, *p* = 0.008**
Pain intensity during attacks [0–10]	9.5 ± 1.1 (19)	**−1.2 ± 2.0 (19)** **d_z_ = 0.61** **Z = −2.8, *p* = 0.006**	−0.9 ± 1.5 (12) d_z_ = 0.58 Z = −2.03, *p* = 0.042	−1.0 ± 1.8 (7) d_z_ = 0.57 Z = −1.6, *p* = 0.11

Note: Values are mean ± SD. Number of patients for each analysis is indicated in parenthesis. Results of pairwise comparison between baseline and the respective period, using the Wilcoxon test are shown. Results that remained significant after Bonferroni-Holm correction for three comparisons are marked in bold. Cohen’s d for pairwise comparisons (d_z_) is given.

**Figure 1.**
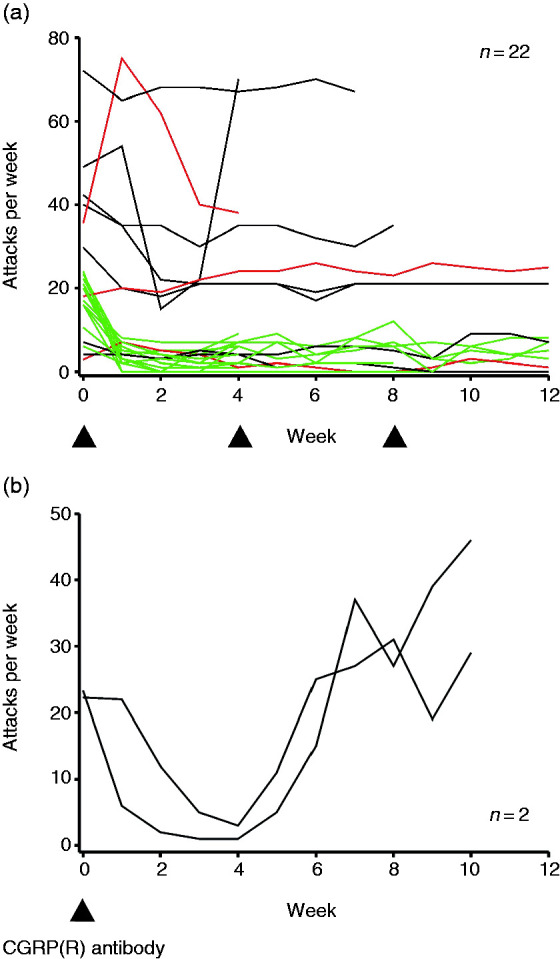
Illustration of individual attack frequencies under CGRP(R) antibody treatment. (a) Illustration of individual attack frequencies under continued CGRP(R) antibody treatment. Green: 50% responders (patients with a ≥50% reduction in attack frequency during the first month); red: patients who had an increase in attack frequency during the first month; black: all remaining patients. (b) Individual attack frequencies in two patients who received and responded to a single injection of a CGRP(R) antibody, illustrating deterioration of attack frequency starting from week 5 after treatment. Arrow heads mark approximate time points of administration of CGRP(R) antibody.

Number of attacks per week at baseline and during ongoing treatment with CGRP(R) antibodies are illustrated in Figure 1(a) for each patient.

Only one patient reported an adverse event after CGRP(R) antibody treatment (fatigue on day 1 after the first injection). This patient had been treated with galcanezumab, and had provided data for the first cycle only.

### The first month after administration of a CGRP(R) antibody

The average number of attacks per week significantly decreased from 23.3 ± 16.4 at baseline to 14.2 ± 18.8 in the first month of treatment with a CGRP(R) antibody (primary outcome, Z = −3.3, *p* < 0.001, Table 3). The average number of applications of acute headache medication per week significantly decreased from 16.2 ± 9.9 at baseline to 6.4 ± 6.9 in the first month of treatment (n = 19, Z = −3.74, *p* < 0.001, secondary outcome). In addition, there was a small but significant decrease in pain intensity during the attacks measured on the NRS (0 − 10) from 9.5 ± 1.1 to 8.3 ± 2.3 (n = 19, Z = −2.76, *p* = 0.006, secondary outcome).

**Figure 2.**
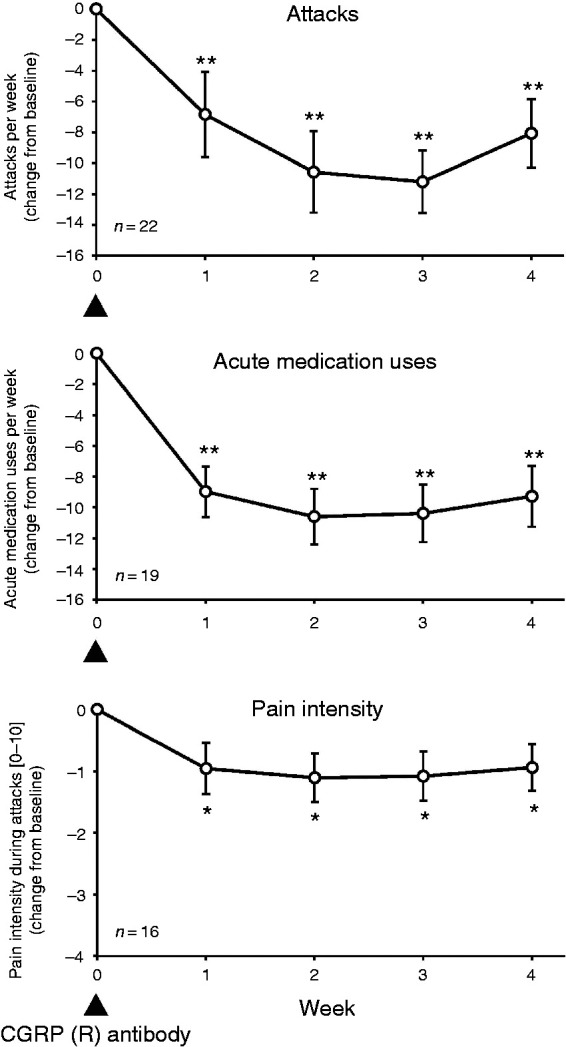
Cluster headache outcomes in the first month of treatment with a CGRP(R) antibody on a weekly basis. Means ± SEM are given. Change from baseline is illustrated. *p < 0.05, **p < 0.01, in the pairwise Wilcoxon test (Bonferroni-Holm corrected for four comparisons). See Table 3 for detailed statistics. Arrow heads mark approximate time points of administration of CGRP(R) antibody.

To better evaluate the onset of treatment effect, we performed a weekly analysis of the first 4 weeks against baseline (Figure 2). For number of attacks per week, there was a significant main effect of time (baseline, week 1, week 2, week 3, week 4: F[4,18] = 8.0, *p* < 0.001). *Post-hoc* tests showed significant differences between baseline and each of the 4 weeks (all corrected *p* < 0.01). For number of weekly applications of acute medication, there was also a main effect of time (F[4,15] = 21.3, *p* < 0.001) and significant *post-hoc* tests for the comparison between baseline and each of the 4 weeks (all corrected *p* < 0.01). Also for pain intensity during attacks, there was a significant main effect of time (F[4,12] = 6.0, *p* = 0.016) and significant differences between baseline and each of the 4 weeks (all corrected *p* < 0.05). These results show that a significant treatment effect was present starting from week 1.

### Responders to CGRP(R) antibody treatment and factors associated with response

Twelve of the 22 patients (55%) were 50% responders; that is, they had a reduction in attack frequency of ≥50% during the first month of treatment with a CGRP(R) antibody (see also Figure 1(a)). A reduction of attack frequency of ≥75% was noted in eight of 22 patients (36%). Three patients experienced an increase in their attack frequency (to 118, 151 and 152% of baseline).

Age, gender, total number of previous preventive treatments, and number of attacks per week at baseline were not significantly associated with response to CGRP(R) antibody treatment (Table 4). Duration of cluster headache in years showed a significant association (longer duration, better response), which however disappeared after correction for multiple comparisons (Table 4). Numbers of subjects treated with galcanezumab (n = 16) vs. erenumab (n = 6) were too small for a meaningful statistical comparison (nominally, one of six erenumab patients and 11 of 16 galcanezumab patients were 50% responders).

**Table 4.** Associations with response to CGRP(R) antibody treatment.

	Number of attacks in month 1 in percent of baselineGroup means ± SD	Statistics
Age	–	rho = 0.08, *p* = 0.797
Gender	Female (15): 51 ± 43%Male (7): 63 ± 52%	Z = −0.46, *p* = 0.680
Duration of cluster headache	–	rho = −0.50, *p* = 0.018, *p*_corr_ = 0.09
Total number of previous preventive treatments	–	rho = −0.05, *p* = 0.819
Number of attacks per week at baseline	–	rho = 0.06, *p* = 0.793

Note: Spearman’s rho and Mann-Whitney U test were used to test for significant associations with the number of attacks during month 1 of treatment expressed in percent of baseline (i.e. smaller percentage, better response). None of the results were significant after Bonferroni Holm correction for five tests (*p*_corr_ indicates the corrected *p*-value).

### Months 2 and 3 during continued administration of CGRP(R) antibody

Data were available for 14 and nine patients for month 2 and month 3, respectively. Comparisons between baseline and months 2 and 3 are shown in Table 3. Number of attacks and use of acute medications were significantly reduced compared to baseline in both months 2 and 3, while reduction of pain intensity during attacks did not reach significance. However, effect sizes (included in Table 3) were similar between months 1, 2 and 3 for reduction of pain intensity.

### Development after cessation of therapy

Two of the 22 patients provided headache diary data before and up to 10 weeks after receiving a single dose of CGRP(R) antibody, to which they had responded. In both, treatment was not immediately continued due to cost coverage issues. Both showed deterioration of attack frequency after week 4, reaching baseline levels between week 6 and 7 (Figure 1(b)).

## Discussion

The main result of the present case series is that attack frequencies of CCH patients were significantly reduced in the first month after administration of a CGRP(R) antibody. Fifty-five percent of the patients were 50% responders. This shows that treatment attempts with CGRP(R) antibodies are successful in an important number of CCH patients with insufficient response to other treatments, and provide a rationale to make these treatments accessible for highly disabled CCH patients on an individual basis.

This proportion of 50% responders (55%) was similar to what has been reported for episodic migraine patients treated with CGRP(R) antibodies (16,17), and larger than what was found in the randomised controlled trial (RCT) on galcanezumab in CCH (33%) (15). The mean reduction of weekly attack frequency in month 1 was also superior in our sample (−9.2) compared to the RCT (∼−4.1) (15), which might in part be due to a larger number of weekly attacks at baseline in the present case series (23.3) versus the RCT (18.8). Further differences to the RCT are the gender ratio (female preponderance in the present study, see below), the number of patients treated with a CGRP(R) antibody (22 vs. 117) and of course the lack of a placebo group in the present study. It must be considered that the placebo effect may be larger during open-label treatment than in placebo-controlled studies, where patients know they may receive placebo. Therefore, results of the present study cannot be taken as proof of the preventive effect of CGRP(R) antibodies under controlled conditions, but show that a considerable proportion of CCH patients insufficiently responding to other treatments responded to CGRP(R) antibodies under real-world conditions.

The present results on attack frequency were corroborated by a reduction in weekly uses of acute attack medication. Pain intensity during attacks was also significantly reduced. However, for pain intensity both statistical effect sizes (see Table 3) and clinical effect sizes (−1.2 points on the NRS [0–10]) were smaller than for attack frequency. Our data suggest that CGRP(R) antibody treatment preferentially acts on attack frequency, with a smaller (and maybe not clinically significant) effect on pain intensity.

It should be noted that the patients in our case series were highly refractory to other preventive treatments, with a documented use of 2–11 preventive treatments previous to the CGRP(R) antibody. Nineteen of the 22 patients fulfilled the criteria for refractory CCH as defined in (8). This makes the present positive results even more important, especially as stimulation of the sphenopalatine ganglion, an invasive procedure which has been specifically tested in refractory CCH patients, is currently not available on the market (18).

The onset of the response to CGRP(R) antibody treatment was within 1 week in the present case series. This is similar to what has been reported for the onset of action of CGRP(R) antibodies in migraine (19,20). Also the CCH RCT on galcanezumab had suggested a rapid onset of action within weeks 1 and 2, which was the only time point significantly different from placebo (15).

The present data suggest that in patients being treated up to 3 months, the mean effect on cluster headache is maintained during this period. Although pain intensity during the attack did not show a significant difference from baseline at months 2 and 3, effect sizes were similar to previous months. Therefore, the lack of statistical significance might be due to the lower numbers in months 2 and 3. However, this should be confirmed in a larger case series.

The data from two patients who, in spite of a good response, were treated for only 1 month (Figure 1(b)) suggest that the effect of CGRP(R) antibody treatment may rapidly decline starting from week 5 (this result is purely exploratory and has to be confirmed in larger samples). It is not known if this would be different after several months of continued treatment. In migraine, a rather gradual decline of effect has been reported after discontinuation of a 6–12 month CGRP(R) antibody treatment (21,22).

We also tested if demographic or cluster headache characteristics were associated with treatment response. No such association was found for age, gender, duration of cluster headache in years, total number of previous preventive treatments, and number of attacks per week at baseline. This is similar to the results of the galcanezumab RCT, which also did not identify any interaction with age, sex, or baseline attack frequency (15). The number of patients treated with erenumab (n = 6) vs. galcanezumab (n = 16) was too small to derive a meaningful comparison between substances. This will have to be analysed when more cases become available. While this manuscript was under review, a case series reporting five cluster headache patients treated with erenumab because of concomitant migraine was published (23). All five patients had an improvement of their cluster headache, but only after 3 months of treatment with erenumab at the high dose (140 mg), so maybe our case series underestimated the effect of erenumab.

It has been suggested that CGRP might be less important in chronic compared to episodic cluster headache, based on several arguments that need to be discussed critically. First, the galcanezumab RCT was significant for episodic but not for chronic cluster headache. This seems in part due to a large placebo or regression to the mean effect (14,15). Second, i.v. CGRP administration induces cluster headache attacks more easily in patients with episodic cluster headache within bout than in CCH (12). However, a more differentiated analysis suggests that this may be related to the inclusion of CCH patients with a low disease activity (quantified by spontaneous attack frequency) in the sample. Third, peripheral blood CGRP levels were higher in episodic compared to chronic cluster headache patients (24). This result must be regarded with some caution as the reliability of CGRP measurement in antecubital vein blood in headache disorders has been critically discussed (see e.g. (25)). The present and previous (23) data showed efficacy of CGRP(R) antibody treatment in CCH under real-world conditions, suggesting a role of CGRP at least in part of the CCH patients. To our knowledge, there is no open-label episodic cluster headache case series our data could be directly compared to. In comparison to the episodic cluster headache RCT, the reduction of weekly attacks was similar (−9.2 vs. −8.7) but the 50% responder rate was smaller in our case series (55%) than in the RCT (71%) (14). Further studies will be needed to evaluate the relative role of CGRP in episodic compared to chronic cluster headache.

Similar to previous reports (e.g. (15)), tolerability of CGRP(R) antibody treatment was good in the present cohort, with only one patient reporting an adverse event (fatigue on the day after injection). It must be noted that patients were not specifically questioned for injection site reactions.

### Strengths and limitations

One important strength of the present analysis is that it is based on headache diary data, which allowed us to use weekly frequency of attacks as the primary endpoint, as recommended in the IHS guideline (26). The same endpoint has been used in the cluster headache galcanezumab RCTs (14,15). It is a drawback that we did not have data on attack severity and use of acute medication for every patient, and that we did not assess patient-reported outcomes such as quality of life. The number of patients in the present case series was limited, especially in months 2 and 3. In addition, in the galcanezumab RCTs on cluster headache, a dose of 300 mg monthly was used (14,15). Since 300 mg are not available on the European market, patients in the present case series were treated with 240 mg. It is not known if this reduces the treatment effect.

There are several possible sources of bias, all inherent to a retrospective case series.

1. Every treating physician decided about CGRP(R) antibody treatment according to his/her clinical judgement, and maybe also based on the availability of the treatment (in the form of free samples and/or cost coverage by the insurance). However, most patients in the present case series were refractory to other preventive treatments, suggesting that this may have been a common requirement for treatment with CGRP(R) antibodies.
2. The use of different headache diaries may have introduced bias. However, all centres used very simple headache diaries. The common denominator was assessment of daily attack frequency, and some additionally collected data on attack severity and use of acute medication.
3. Substances and doses used and treatment duration were heterogeneous.
4. Four patients treated with a CGRP(R) antibody were excluded as they did not provide documentation of their headache attacks. This may have introduced a bias, as patients not documenting are often those who are severely affected and have a long history of cluster headache. However, obtaining headache diary data from 82% (22/26) of the treated patients seems satisfactory for a case series.
5. Patients stopping treatment (or documentation) for lack of effect has the potential to bias results in later months, so results from months 2 and 3 should be regarded with caution.
6. The present data stem from open-label treatment. Expectations may have potentiated the treatment effect. Significant placebo effects have been seen in cluster headache (14,15). On the other hand, most of our patients were refractory to other preventive treatments. In the migraine studies on CGRP(R) antibodies, it has been repeatedly reported that treatment refractory patients tend to have less placebo effect than naïve patients (27,28).
7. Fifteen of the 22 patients in the present case series were female. This is in contrast to typical sex ratios in CCH, reported to be between 2.6:1 and 4.7:1 (men:women) (29,30). One reason may be that in Europe, CGRP(R) antibodies are approved for treatment of migraine but not of cluster headache. Migraine is more frequent in females, and indeed part of the patients received CGRP(R) antibody treatment because of comorbid migraine. In addition, females may be more severely affected by cluster headache than males, having more and longer attacks and less response to acute therapy (31,32), possibly leading to an overrepresentation among off-label treated patients. Moreover, all four patients who could not be included because of lack of documentation were male, leading to an additional shift in sex distribution. However, the average treatment effect was similar in males and females in the present analysis (Table 4).
8. One patient taking prednisolone adapted his daily dose between 10 and 75 mg according to attack frequency and severity. This patient was a responder to galcanezumab. He had taken prednisolone as needed for 6 months previous to starting galcanezumab, and had been able to reduce his daily dose of prednisolone from 40–75 mg before galcanezumab to 10 mg after the second dose of galcanezumab.
9. The number of attacks in one patient (72 attacks/week at baseline) exceeded the upper limit of 8/day stated in the ICHD-3 criteria (4). The diagnosis of cluster headache in this patient has been independently confirmed by two tertiary care headache centres. However, this patient may not be representative for chronic cluster headache patients in general.

## Conclusion

The present case series shows that under real-world conditions, 55% of our 22 CCH patients responded to CGRP(R) antibody treatment, experiencing a rapid and significant reduction of attack frequency and pain intensity. This supports individual off-label treatment attempts with CGRP(R) antibodies in CCH patients.

## Clinical implications

- Chronic cluster headache is highly disabling and not all patients respond to standard treatment.
- Within our chronic cluster headache case series, 55% were 50% responders to CGRP(-receptor) antibodies, showing the value of individual treatment attempts with these substances.
- These data support attempts to ask health care providers for reimbursement of individual off-label treatment with CGRP(-receptor) antibodies in refractory chronic cluster headache patients.
